# tDCS of the Cerebellum: Where Do We Stand in 2016? Technical Issues and Critical Review of the Literature

**DOI:** 10.3389/fnhum.2016.00199

**Published:** 2016-05-11

**Authors:** Kim van Dun, Florian C. A. A. Bodranghien, Peter Mariën, Mario U. Manto

**Affiliations:** ^1^Clinical and Experimental Neurolinguistics, Vrije Universiteit BrusselBrussels, Belgium; ^2^Unité d'Etude du Mouvement, Laboratoire de Neurologie Expérimentale, Université libre de Bruxelles (ULB)Brussels, Belgium; ^3^Department of Neurology and Memory Clinic, ZNA Middelheim General HospitalAntwerp, Belgium; ^4^Service des Neurosciences, Université de MonsMons, Belgium

**Keywords:** cerebellum, tDCS, tACS, intensity, electrode placement, sham, offline vs. online, anodal vs. cathodal

## Abstract

Transcranial Direct Current Stimulation (tDCS) is an up-and-coming electrical neurostimulation technique increasingly used both in healthy subjects and in selected groups of patients. Due to the high density of neurons in the cerebellum, its peculiar anatomical organization with the cortex lying superficially below the skull and its diffuse connections with motor and associative areas of the cerebrum, the cerebellum is becoming a major target for neuromodulation of the cerebellocerebral networks. We discuss the recent studies based on cerebellar tDCS with a focus on the numerous technical and open issues which remain to be solved. Our current knowledge of the physiological impacts of tDCS on cerebellar circuitry is criticized. We provide a comparison with transcranial Alternating Current Stimulation (tACS), another promising transcranial electrical neurostimulation technique. Although both tDCS and tACS are becoming established techniques to modulate the cerebellocerebral networks, it is surprising that their impacts on cerebellar disorders remains unclear. A major reason is that the literature lacks large trials with a double-blind, sham-controlled, and cross-over experimental design in cerebellar patients.

## Introduction

During the past 15 years a high number of studies have shown that transcranial Direct Current Stimulation (tDCS) is a simple and robust technique to modulate cortical excitability of the human brain (Nitsche and Paulus, 2011; Brunoni et al., 2012). Currently the technique is widely used in healthy subjects with the goal of enhancing both motor and cognitive functions (Reis and Fritsch, 2011; Coffman et al., 2014). tDCS is also applied in various neurological disorders to improve motor, cognitive, and affective deficits (Brunoni et al., 2012; Flöel, 2014). Many researchers have primarily focussed on stimulating cortical regions (e.g., the motor cortex and the prefrontal areas). tDCS is now increasingly used as a tool to stimulate or inhibit the cerebellar circuitry (Ferrucci et al., 2015).

One of the particularities of the cerebellum is that it holds the highest concentration of neurons of the brain. Although the entire cerebellum only represents 10% of the whole brain volume it contains likely more than 80% of its neurons (Herculano-Houzel, 2009). As tDCS mainly acts on neurons and given the anatomical organization of the cerebellum immediately below the skull, tDCS is particularly interesting for an effective neuromodulation of the cerebellar circuits. Since the cerebellum is closely connected to the cerebrum via closed parallel loops that reciprocally link the cerebellum with both motor and associative cortical areas, cerebellar stimulation may functionally affect cerebellocerebral interactions and modulate functions residing elsewhere in the brain (Grimaldi et al., 2014a; Priori et al., 2014). Indeed, a number of recent studies have shown that tDCS induces significant changes in cerebellar excitability (Ferrucci et al., 2015). Table 1 presents an overview of studies using cerebellar tDCS in both healthy and neurological populations. The available literature on tDCS studies focussing on the cerebellum was identified through searches of electronic online databases (*Web of Knowledge, ScienceDirect, PubMed, Medline*), using the following keywords in Boolean search: cerebell* AND tDCS OR transcranial direct current stimulation. This search generated 84 articles, of which 43 were selected after careful reading of the abstract by the first author. Bibliographies of all relevant articles were scanned to identify additional references. Only original studies using tDCS with one electrode on the cerebellum were included in this review.

**Table 1:** **Overview of studies using cerebellar tDCS**.

**Study**	**Number of participants**	**Participants**	**Type of tDCS**	**Online/offline**	**mA**	**1 session**	**Number of sessions**	**Position of active electrode**	**Position of reference electrode**	**Domain studied**
Ferrucci et al., 2008	17	Healthy	Anodal/cathodal/sham	Offline	2 mA	15 min	1 anodal/1 cathodal/1 sham (1 week apart)	2 cm below inion, 1 cm posterior to mastoid process	Right deltoid muscle	Working memory
Galea et al., 2009	16 (10 M, 6 F)	Healthy	Anodal/cathodal/sham	Offline	2 mA	25 min	1 anodal/1 cathodal/1 sham (6 days apart)	3 cm right to inion	Right buccinator muscle	Cerebellar excitability
Galea et al., 2011	72 (38 M, 34 F)	Healthy	Anodal/sham	Online	2 mA	~15 min	1 session (anodal or sham)	3 cm right to inion	Right buccinator muscle	Motor adaptation
Ferrucci et al., 2012	21 (9 M, 12 F)	Healthy	Anodal/cathodal/sham	Offline	2 mA	20 min	1 anodal/1 cathodal/1 sham (1 week apart)	2 cm below inion, 1 cm medially to mastoid apophisis	Right deltoid muscle	Facial emotion recognition
Jayaram et al., 2012	40 (25 M, 15 F)	Healthy	Anodal/cathodal/sham	Online	2 mA	15 min	1 session (anodal, cathodal, or sham)	3 cm lateral to inion (ipsilateral to fast or to slow leg)	Ipsilateral buccinator muscle	Locomotor adaptation
	8 (5 M, 3 F)	Healthy	Anodal/cathodal/sham	Online	2 mA	5 min	1 session (anodal, cathodal, or sham)	3 cm lateral to inion (ipsilateral to fast or to slow leg)	Ipsilateral buccinator muscle	Baseline walking
	5 (2 M, 3 F)	Healthy	Anodal	Online	2 mA	20 min	1 session (anodal, cathodal, or sham)	3 cm lateral to inion (ipsilateral to fast or to slow leg)	Ipsilateral buccinator muscle	Walking trajectory
Pope and Miall, 2012	66 (12 M, 54 F)	Healthy	Anodal/cathodal/sham	Offline	2 mA	20 min	1 session (anodal, cathodal, or sham)	1 cm below, 4 cm right to inion	Right deltoid muscle	Working memory
Block and Celnik, 2013	79 (32 M, 47 F)	Healthy	Anodal/sham	Online	2 mA		1 session (anodal or sham)	3 cm right or left to inion	Ipsilateral buccinator muscle	Visuomotor adaptation
Boehringer et al., 2013	40 (20 M, 20 F)	Healthy	Cathodal/sham	Offline	2 mA	25 min	1 cathodal/ 1 sham (min 5 days apart)	2 cm below inion, 1 cm posterior to right mastoid process	Right buccinator muscle	Verbal working memory
Ferrucci et al., 2013	21 (9 M, 12 F)	Healthy	Anodal/sham	Offline	2 mA	20 min	1 anodal/ 1 sham (1 week apart)	bilateral: 2 cm below inion, 1 cm medially to mastoid apophysis	Right arm	Procedural learning
Foerster et al., 2013	18 (2 M, 16 F)	Healthy	Anodal/sham	Online	2 mA	13 min	1 anodal/1 sham (48 h apart)	3 cm right to inion	Right deltoid	Motor performance
Grimaldi and Manto, 2013	9 (7 M, 2 F)	Core cerebellar syndrome	Anodal/sham	Offline	1 mA	20 min	1 sham/1 anodal	3 cm right to inion	Contralateral supra-orbital area	Coordination and stretch reflexes
	9 (7 M, 2 F)	Core cerebellar syndrome	Anodal/sham	Offline	1 mA	20 min	1 sham/1 anodal	bilateral: in front of vermis at inion level	Contralateral supra-orbital area	Posture
Sadnicka et al., 2013	12 (9 M, 3 F)	Healthy	Anodal/cathodal/sham	Offline	2 mA	15 min	1 cathodal/1 anodal/1 sham (1 week apart)	3 cm right to inion	Ipsilateral buccinator muscle	Motor surround inhibition
Shah et al., 2013	8 (5 M, 3 F)	Healthy	Anodal/cathodal	Online	1 mA	15 min	1 anodal /1 cathodal (min 96 h apart)	3 cm left to inion	Ipsilateral buccinator muscle	Motor learning
Bradnam et al., 2014 (letter to the editor)	1 F	Cervical dystonia	Anodal	Offline	2 mA	2 × 15 min (5 min apart)	2 sessions/week (12 weeks)	3 cm right or left to inion	Ipsilateral buccinators	Cervical dystonia
Chen et al., 2014	10 (4 M, 6 F)	Healthy	Anodal/cathodal/sham	Offline	2 mA	25 min	1 anodal/1 cathodal/1 sham (min 7 days apart)	3 cm right to inion	Right buccinator muscle	Somatosensory mismatch negativity
	10 (8 M, 2 F)	Healthy	Anodal/cathodal/sham	Offline	2 mA	25 min	1 anodal/1 cathodal/1 sham (min 7 days apart)	3 cm right to inion	Right buccinator muscle	Auditory mismatch negativity
Dutta et al., 2014	12 (M)	Healthy	Anodal/sham	Offline/online	1 mA	15 min	Offline and online tDCS (1 week apart)	3 cm left to inion	Right supraorbital ridge (forehead)	Myoelectric control
Gironell et al., 2014 (letter to the editor)	10 (6 M, 4 F)	Essential tremor	Cathodal/sham	Offline	2 mA	20 min	5 sessions/week (2 weeks)	3 cm left and right to inion (2 cathodes)	Fp1 and Fp2 (2 anodes)	Essential tremor
Grimaldi et al., 2014b	2 (1 M, 1 F)	Cerebellar ataxia	Anodal/sham	Offline	1 mA	20 min	1 session (sham and anodal, always first sham)	3 cm right to inion	Contra-lateral supraorbital area	Upper limb tremor
Hardwick and Celnik, 2014	22 (11 M, 11 F)	Healthy, old	Anodal/sham	Online	2 mA	~15 min	1 session (anodal or sham)	3 cm lateral to inion; ipsilateral to dominant hand	Ipsilateral buccinator muscle	Motor adaptation
Herzfeld et al., 2014	37	Healthy	Anodal/cathodal/sham	Online	2 mA	25 min	1 session (anodal, cathodal, or sham)	3 cm right to inion	Right buccinator muscle	Acquisition and retention of motor memories
Ho et al., 2014	7	Major depressive disorder	Cathodal	Offline	2 mA	20 min	5 sessions/week (4 weeks)	bilateral:centered over inion	Left supraorbital region	Depression
Macher et al., 2014	16 (8 M, 8 F)	Healthy	Anodal/cathodal/sham	Offline	2 mA	25 min	1 anodal/1 cathodal/1 sham (1 week apart)	2 cm below inion, 1 cm posterior to right mastoid process	Right buccinator muscle	Verbal working memory
Zuchowski et al., 2014	30 (12 M, 18 F)	Healthy	Anodal/cathodal/sham	Online	2 mA		1 session (anodal, cathodal, or sham)	3 cm right to inion	Ipsilateral buccinator muscle	Conditioned eye-blink responses
Avila et al., 2015	13 (7 M, 6 F)	Healthy	Anodal/sham	Online	1.5 mA	15 min	1 anodal/1 sham (3–7 days apart)	3 cm right to inion	Left buccinator muscle	Eye saccade adaptation
Benussi et al., 2015	19 (8 M, 11 F)	Cerebellar ataxia	Anodal/sham	Offline	2 mA	20 min	1 anodal/1 sham (1 week apart)	bilateral: 2 cm below inion, lateral borders 1 cm medially to mastoid apophysis	Right deltoid muscle	Ataxia
Bersani et al., 2015	27 (10 M, 17 F)	Bipolar disorder type I or II	Cathodal	Offline	2 mA	20 min	5 sessions/week (3 weeks)	1 cm below, 4 cm right to inion	Left dorsolateral prefrontal cortex (Fp1)	Neurophysiological performance
Bocci et al., 2015	15 (8 M, 7 F)	Healthy	Anodal/cathodal/sham	Offline	2 mA	20 min	1 anodal/1 cathodal/1 sham (1 week apart)	bilateral: 2 cm below inion, lateral borders 1 cm medially to mastoid apophysis	Right shoulder	Nociceptive perception
Bradnam et al., 2015	8 dystonic (7 M, 1 F); 8 controls (6 M, 2 F)	5 writer's cramp; 3 musician's cramp	Anodal/cathodal/sham	Offline	2 mA	20 min	1 anodal/1 cathodal/1 sham (min 5 days apart)	1 cm below, 3 cm lateral to inion	Right buccinator muscle	Dystonia
Calzolari et al., 2015	1 M	Bilateral occipital + left cerebellar damage (mild left spatial neglect)	Anodal/sham	Online	1.5 mA	15 min	1 left/1 right/1 sham (~ 91 h apart)	2 cm below inion, 1 cm medially to right or left mastoid process	Ipsilateral deltoid	Prism adaptation
Cantarero et al., 2015	33 (13 M, 20 F)	Healthy	Anodal/cathodal/sham	Online	2 mA	~20 min	1 anodal/1 cathodal/1 sham (3 consecutive days)	3 cm right to inion	Right buccinator muscle	Motor skill learning
Doeltgen et al., 2015	14 (5 M, 9 F)	Healthy	Anodal/sham	Offline	2 mA	20 min	1 anodal/1 sham (min 5 days apart)	1 cm below, 3 cm right to inion	Ipsilateral buccinator muscle	Functional connections
	13 (7 M, 6 F)	Healthy	Anodal/sham	Offline	2 mA	20 min	1 anodal/1 cathodal/1 sham (min 5 days apart)	1 cm below, 3 cm right to inion	Ipsilateral forehead	Functional connections
Martin et al., 2015	15	Bipolar disorder	Cathodal/sham	Online	2 mA	30 min	1 cathodal/1 sham (1 week apart)	bilateral: central over the inion	Left F3	Working memory
Minichino et al., 2015	27	Bipolar disorder type I or II	Cathodal	Offline	2 mA	20 min	5 sessions/week (3 weeks)	1 cm below, 4 cm right to inion	Left DLPFC	Neuropsychological functioning
Panouillères et al., 2015a	80 (older: 18 M, 20 F; younger: 22 M, 20 F)	Healthy	Anodal/sham	Online	2 mA	17 min	1 session (anodal or sham)	3 cm right to inion	Left superior aspect of trapezius	Motor adaptation
Panouillères et al., 2015a	79 (35 M, 44 F)	Healthy	Anodal/cathodal/sham	Online	2 mA	25 min	1 session (anodal, cathodal, or sham)	bilateral: centered over inion	Superior aspect of right trapezius muscle	Saccadic forward and backward adaptation
Picazio et al., 2015	13 (6 M, 7 F)	Healthy	Anodal/cathodal/sham	Offline	2 mA	20 min	1 anodal/1 cathodal/1 sham (1 week apart)	1 cm below, 3 cm left to inion	Left deltoid	Musical and spatial information processing
Wessel et al., 2016	38 (17 M, 21 F)	Healthy	Anodal/cathodal/sham	Online	2 mA	20 min	1 session (anodal, cathodal, or sham)	3 cm right to inion	Right buccinator muscle	Temporal motor skill
Yavari et al., 2016	29 (12 M, 17 F)	Healthy	Anodal/cathodal/sham	Online	2 mA	15 min	1 session (anodal, cathodal, or sham)	3 cm right to inion	Right buccinator	Visuomotor adaptation
Bation et al., 2016	8	Treatment-resistant OCD	Anodal	Offline	2 mA	20 min	2 sessions/day (5 days)	3 cm below inion, 1 cm right from midline	Left OFC (Fp1)	OCD
Bocci et al., 2016	16 (7 M, 9 F); 16 controls	Highly hypnotizable	Anodal/cathodal	Offline	2 mA	15 min	1 session (anodal or cathodal)	bilateral: 2 cm below inion, lateral borders 1 cm medially to mastoid apophysis	Right shoulder	Nociceptive perception
Chothia et al., 2016	12 (7 M, 5 F)	Healthy	Anodal/sham	Offline	2 mA	15 min	1 anodal/1 sham (min 5 days apart)	3 cm left to inion	Left buccinator muscle	Motor adaptation
Ferrucci et al., 2016	9 (5 M, 4 F)	Idiopathic PD	Anodal/sham	Offline	2 mA	20 min	5 sessions/week (1 week): 1 anodal/1 sham (1 month apart)	Bilateral: 1–2 cm below inion, lateral borders ~1 cm medially to mastoid apophysis	Right shoulder	Levodopa-induced dyskinesia
Van Wessel et al., 2016	12 (6 M, 6 F)	Healthy	Anodal/cathodal/sham	Online	2 mA	20 min	1 anodal/1 cathodal/1 sham (min 5 days apart)	3 cm right to inion	Left buccinator muscle	Working memory

Although the application of tDCS in experimental and clinical settings is exponentially growing, little is known about the specific mechanisms by means of which tDCS modulates motor, cognitive, and affective functions. A consensus exists about the mechanisms of action in cerebral tDCS (Horvath et al., 2015), but since the cerebellum has an entirely different cytoarchitecture than the neocortex, generalizations of the findings obtained in the studies based upon cerebral tDCS are hard to make (Schlerf et al., 2014). A number of recent studies have indeed shown that cerebellar cell morphology might have a great impact on the polarity-dependent excitability changes and on the effectiveness of the stimulation (Rahman et al., 2014). Moreover, the complex cerebellar folding influences the direction of the current relative to the cell morphology and as a result affects the magnitude and the polarity of the somatic membrane potential changes making it difficult to predict the outcome of cerebellar stimulation (Rahman et al., 2014). As a result, the exact mechanisms subserving tDCS remain to be clearly identified. The effects of stimulation duration, of the number and frequency of sessions, of the intensity of the current, and of the placement of the electrodes have not been systematically investigated for the cerebellum (Ferrucci et al., 2015). In one modeling study cell morphology of the cerebellum was taken into account to theorize about the functional effect of a polarizing current on the different zones of the cerebellum (Rahman et al., 2014).

This article aims to present a concise overview of the different methods of cerebellar tDCS that are currently used and summarizes our current knowledge about the physiological impact of tDCS on cerebellar neurons. A number of guidelines for the different parameters to safely and reliably apply cerebellar tDCS are discussed as well. Finally, a short comparison with transcranial Alternating Current Stimulation (tACS), another emerging tool, is made.

## Technical issues

### Electrode placement and modeling studies

**Figure 1:**
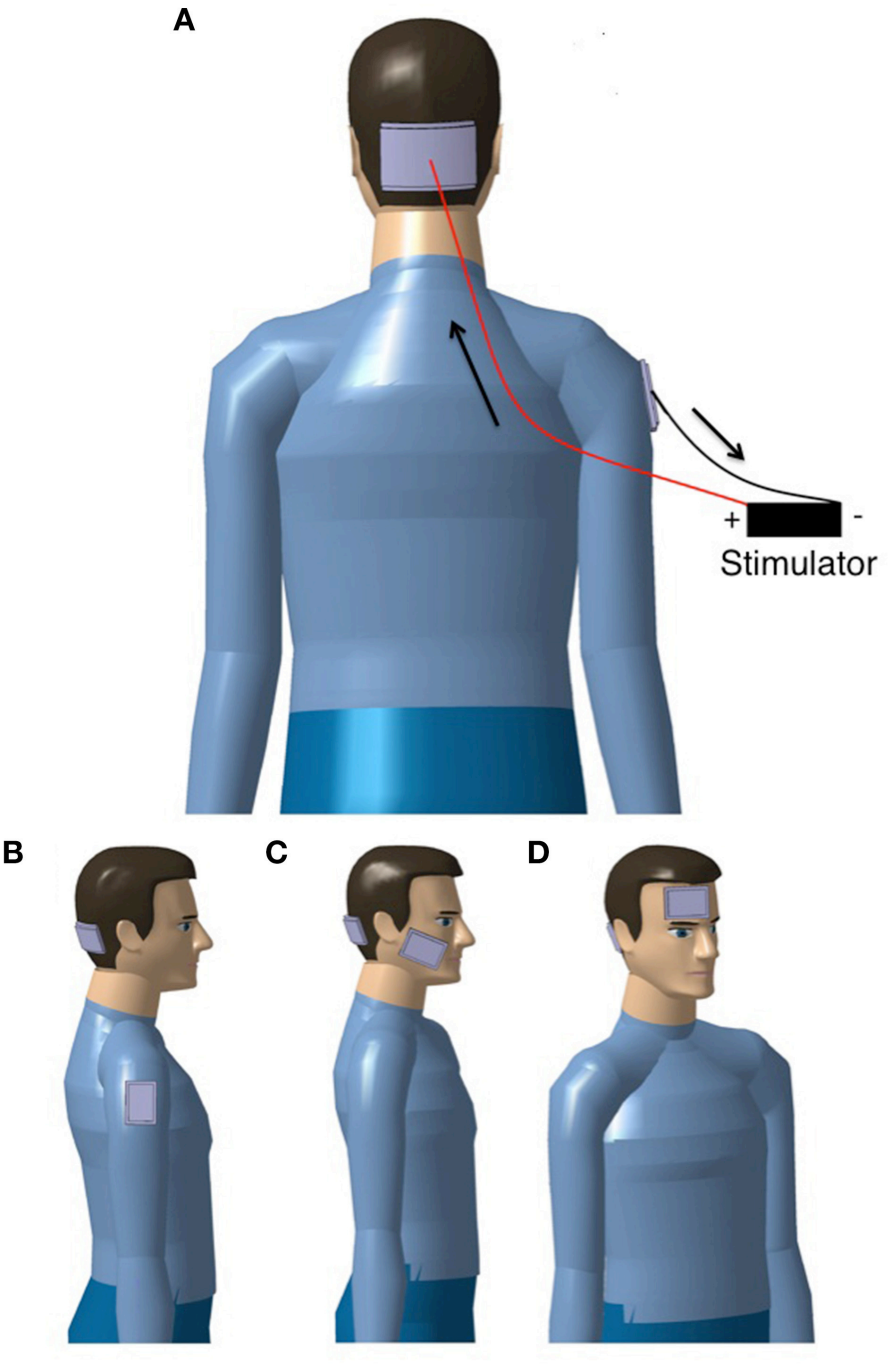
**Examples of set-ups to apply tDCS over the cerebellum. (A)** bilateral setup aiming to stimulate simultaneously the two cerebellar hemispheres and the vermis, the flow of the current is indicated with arrows for **anodal** stimulation of the cerebellum (for cathodal stimulation, the flow of the current is reversed); **(B–D)** unilateral setups (the target is one cerebellar hemisphere) with the reference electrode over **(B)** the deltoid muscle, **(C)** the buccinator muscle, and **(D)** the forehead/supraorbital area. For simplicity, the wires and stimulator are only shown in **(A)**.

The most frequently used placement of electrodes in studies on cerebellar tDCS is a lateralized position with the active electrode placed on the skin over one cerebellar hemisphere at 1–2 cm below and 3–4 cm lateral to the inion (Ferrucci et al., 2015) and the reference electrode over (a) the ipsilateral buccinator muscle, (b) the ipsilateral deltoid muscle, or (c) the ipsilateral forehead/supraorbital area (see Figure 1). Although most unilateral cerebellar stimulation setups made use of an ipsilateral reference electrode, some placed the reference electrode contralaterally. The possible differences in terms of effects between both setups have not been investigated yet. Bilateral stimulation of the cerebellum is also possible, but for bilateral stimulation the setups differ substantially (see Table 1 for the electrode placements in each study). It is assumed that a bilateral stimulation of the cerebellum would impact on both cerebellar hemispheres with a more diffuse effect upon the cerebral cortex. Stimulation of the vermis requires a placement of the active electrode in the midline.

Since electrodes' positions determine the direction of the current flow and the orientation of the electric field (Ferrucci et al., 2015), modeling studies have attempted to estimate the current density field distributions and electric fields induced in the nervous tissue by tDCS using computational methods to solve the Laplace equation (Priori et al., 2014). However, only a few modeling studies have specifically investigated the current flow in cerebellar tDCS (Parazzini et al., 2014b; Priori et al., 2014; Rahman et al., 2014; Rampersad et al., 2014). Parazzini et al. (2014b) modeled cerebellar tDCS using three virtual human head models of different ages and genders constructed of 77 different tissue types, segmented into a (hexahedral) voxel-based format (1 mm voxels). In this study a bilateral setup was used with the active electrode centered on the median line, 2 cm below the inion, and the reference electrode over the right arm (5 × 7 cm). A current intensity of 2 mA was used. The authors found that the highest electric field and current density was located below the stimulating electrode in the posterior cerebellum. Only a slight spread to other structures (e.g., occipital cortex) was found, unlikely sufficient to produce relevant functional effects. Parazzini et al. (2014b) also showed that no alteration of brainstem excitability occurred (this is particularly important given the numerous connections between brainstem nuclei and the cerebellum) and that there was only a very low current spread to the heart. There are, however, some slight differences between the models. In particular, differences in cerebrospinal fluid (CSF) distribution and/or skull thickness may influence the spread of the field amplitude toward the occipital region. Although Parazzini et al. (2014b) did not find a significant spread of the current to the brainstem in the child model, the use of cerebellar tDCS in children is still discouraged due to a possible spread of the current to this area (Priori et al., 2014; Ferrucci et al., 2015). Since the direction of the field within the cerebellum was not addressed in this study, Rahman et al. (2014) investigated this issue using four different electrode montages, varying the direction of the current flow (inward, outward, lateralized left, lateralized right) while only stimulating the cerebellar area. They found that in the four simulated setups, a current flow was induced and was largely uniform in direction, confirming the findings of Parazzini et al. (2014b) that the cerebellum can indeed be stimulated with cerebellar tDCS (Rahman et al., 2014). However, both studies focused on bilateral cerebellar stimulation, a setup that is not commonly used in experimental studies.

Rampersad et al. (2014) investigated six of the most frequently used setups in clinical and experimental cognitive research with finite element models. They used an MRI- and DTI-based model of a healthy 25-year-old man with 11 different tissue types, reconstructed as a mesh of tetrahedral elements. To simulate cerebellar stimulation they placed a square anode of 5 × 5 cm, 3 cm rightwards of the inion and a square cathode of 5 × 5 cm on the right buccinator muscle (cheek). Simulations were made for 1 mA tDCS. Results showed that during cerebellar stimulation, the actual maximum of the electric field is more inferior and medial to the targeted area due to the highly concave shape of the area. However, the high electric field also covered most of the inferior surface of the right cerebellar hemisphere, which makes it the most efficient setup of this modeling study. The study also showed that the maximum electric field strength values are much lower in the cerebellar setup. This is probably due to large amounts of shunting under the skull and through the skin. In all configurations only a small amount of the current enters the brain, but this was especially true for the cerebellar setup since the cerebellar electrode is placed on the back of the head. Most of the remaining current enters the gray matter perpendicularly. This might be more important than the mean or the maximum electric field strength (Rampersad et al., 2014). Overall, this study has validated most experimental setups applied in experiments with cerebellar tDCS.

Although modeling studies provide insights in the understanding of cerebellar tDCS, the results should be interpreted with much caution since little is known about tissue conductivity (Priori et al., 2014). Especially the values of muscle conductivity vary substantially in the literature (Rampersad et al., 2014). In their study, Rampersad et al. (2014) compared the results obtained with the largest values (as reported for the neck muscles) with the lowest reported values and found an increase of 11% in the mean field strength in the target volume. Efforts should be devoted to improve our knowledge about tissue conductivity. This would increase significantly the accuracy of the modeling studies (Rampersad et al., 2014).

It is still unclear whether the position of the reference electrode is critical or not. Grimaldi and Manto (2013), for instance, used a setup with the reference electrode on the contralateral supraorbital area. To exclude the possibility that the results were due to an inhibition of the prefrontal area, they repeated the experiment with the reference electrode on the ipsilateral shoulder. The results remained unchanged. In addition, the model of Parazzini et al. (2014b) showed that varying the position of the active electrode with ±1 cm only induced a small change in the field amplitude distributions, suggesting that the use of advanced neuronavigation systems is probably not needed to reliably perform cerebellar tDCS. The clinical evidence of studies using cerebellar tDCS in different setups seems to corroborate this view. However, more modeling and clinical studies are needed to systematically investigate the impact of electrode placement on the effects induced by cerebellar tDCS (Priori et al., 2014; Ferrucci et al., 2015).

### Stimulation type

There are two types of tDCS that can be used, depending on the direction of the current: anodal and cathodal. Anodal stimulation is frequently associated with enhanced neuronal excitability below the site of stimulation, whereas cathodal stimulation is thought to inhibit neuronal excitability (Rahman et al., 2013). However, this seems to be a simplification of the mechanisms of action. To understand which type of stimulation should be used, it is important to keep in mind the impact of tDCS on neurons.

Neurons, when inactive, remain at their resting electric potential due to the concentration gradient between the intra- and the extra-cellular medium. This electric potential can be estimated using the Goldman-Hodgkin-Katz voltage equation (Hodgkin and Katz, 1949). When tDCS is applied, a difference of electric potential is created between the stimulator's anode and cathode to allow a constant current flow between them. This difference of potential results in an electric field equal to the opposite of the potential gradient (according to Maxwell's law). The electric field induces a shift in the membrane electric potential of the neuron. This potential change can influence neuronal activity but is not strong enough to induce action potentials inside the neurons. As a result, tDCS can only modulate excitability in active neurons and has little or no impact on resting neuronal populations (Woods et al., 2016).

A positive (cathodal) extra-cellular field hyperpolarizes the membrane and lowers the action potential firing rate (i.e., lower excitability), whereas a negative (anodal) extra-cellular field depolarizes the membrane and increases the action potential firing rate (i.e., hyperexcitability; McIntyre and Grill, 1999; Liebetanz et al., 2002; Bikson et al., 2004). However, these physiological mechanisms are not always operational and depend on the orientation of neuronal structures. Whether an electric field has an excitatory or inhibitory effect depends on the axonal orientation relative to the field (parallel vs. perpendicular, current flow from soma to dendrites vs. from dendrites to soma; Kabakov et al., 2012; Rahman et al., 2014). In addition, it remains unclear which compartments (soma, dendrites, axons) are involved in modulation through electrical stimulation and whether depolarization or hyperpolarization is responsible for enhancing synaptic efficacy (Rahman et al., 2013).

tDCS also causes polarity-dependent physiological changes in the neurons that can last for a few hours after the end of a stimulation session (so called after-effects), depending on the intensity and duration of stimulation (Manto et al., 2011). One of the mechanisms that might be responsible for these long-lasting after-effects is a change in the ionic gradient (due to a change in membrane potential) on the extra-cellular side (Ardolino et al., 2005; Priori et al., 2014), or at the synaptic level by N-methyl-D-aspartate (NMDA) receptors (Liebetanz et al., 2002; Ardolino et al., 2005). It is suspected that anodal tDCS may change the intra-cellular Ca^2+^ level, leading to an NMDA receptor-mediated augmentation of synaptic strength, while cathodal membrane hyperpolarization may lead to a depression of synaptic strength (Woods et al., 2016). In addition, it has been shown that an externally applied electric field causes redistribution of membrane proteins and migration of the acetylcholine receptors (Ardolino et al., 2005). Both the protein redistribution and the channel migration may affect the propagation of neuronal activity and change neuronal plasticity (Debanne et al., 2003). tDCS has also been reported to change the acid-base balance due to water electrolysis from constant current. This mechanism may affect membrane, receptor, and cell function (Ardolino et al., 2005). If recommended stimulation duration and intensity are respected, these changes are only temporary and no stable functional or structural cortical modifications have been observed after tDCS (Nitsche et al., 2003b). However, since the physiological effects of electrical stimulation are studied using computer models based on the Hodgkin-Huxley and cable models (McIntyre and Grill, 1999; McIntyre, 2004; Manola et al., 2005; Molaee-Ardekani et al., 2013; Rahman et al., 2013; Dougherty et al., 2014; Parazzini et al., 2014a), novel studies on animals and/or humans are needed to better understand the complex effects of tDCS on brain function.

**Figure 2:**
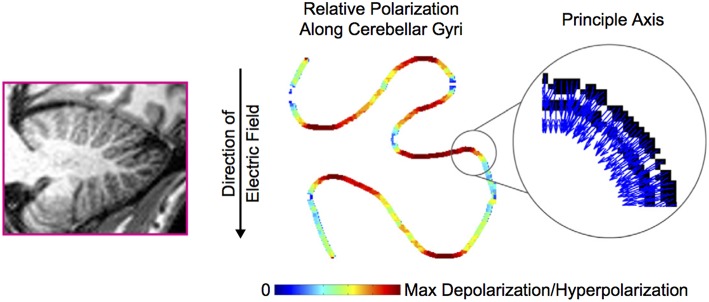
**Cerebellar folding influences polarization along the sulci**. The principal axis of the Purkinje cells (indicated with blue arrows—the color code has no particular significance in the inset) along a trace of cerebellar gyri is subject to an electric field. The resulting polarization (maximal hyperpolarization or depolarization) is indicated in false color along the trace. Adapted from Rahman et al. (2014). With permission from Elsevier.

As expected, the effects of tDCS critically depend on (a) the previous neuronal physiological state and (b) the structure orientation relative to the electric field direction (Bikson et al., 2004; Manola et al., 2005; Woods et al., 2016). Neurons of the cerebellum are not identically orientated and even follow complex anatomical distributions over the numerous folia. This will cause a hyperpolarization in some compartments while others will be depolarized at the same time (Figure 2). Therefore, the global effects of tDCS on the cerebellum remain difficult to simulate (Woods et al., 2016). The linking function of parallel fibers in the cerebellar cortex and the peculiar disposition of the 10 lobules of the cerebellum surrounded by CSF and vessels render the simulation even more difficult.

There is currently a lack of information about the specific effects of tDCS on the various cerebellar neurons (Purkinje neurons, inhibitory interneurons of the cerebellar cortex, granule cells, nuclear neurons) and on the afferent pathways (mossy fibers and climbing fibers). Several studies using cerebellar tDCS have compared both anodal and cathodal stimulation with a sham condition. Varying results were obtained in these studies. Most of the studies reported a different effect for anodal and cathodal tDCS. Chen et al. (2014), Galea et al. (2009), Herzfeld et al. (2014), Jayaram et al. (2012), Yavari et al. (2016), and Zuchowski et al. (2014) reported an increased cerebellar brain inhibition (CBI) following anodal stimulation applied over the cerebellar cortex. By contrast, cathodal stimulation reduced CBI. Only two studies found the reverse effect (Bocci et al., 2015; Panouillères et al., 2015b). The remaining studies found an effect of either cathodal (Pope and Miall, 2012; Picazio et al., 2015) or anodal (Macher et al., 2014; Cantarero et al., 2015; Bocci et al., 2016; Wessel et al., 2016) stimulation. A few studies did not find any effect after both stimulation types (Jayaram et al., 2012; Sadnicka et al., 2013; Chen et al., 2014; Bocci et al., 2016). Heterogeneous effects of cerebellar stimulation in different tasks have been observed. In general, anodal stimulation is believed to enhance motor and cognitive functions, whereas cathodal stimulation typically inhibits functioning. However, in several studies, cathodal stimulation was associated with a neurobehavioral effect in agreement with an enhanced function of the cerebellar cortex (Galea et al., 2011; Ferrucci et al., 2012; Pope and Miall, 2012; Shah et al., 2013; Bersani et al., 2015; Bocci et al., 2015; Bradnam et al., 2015; Minichino et al., 2015; Panouillères et al., 2015b). On the other hand some studies have shown that anodal stimulation may impair cerebellar function (Ferrucci et al., 2008; Foerster et al., 2013; Dutta et al., 2014; Macher et al., 2014; Bocci et al., 2015; Doeltgen et al., 2015; Panouillères et al., 2015b; Chothia et al., 2016). For instance, Panouillères et al. (2015b) showed that cathodal cerebellar tDCS facilitates saccadic adaptation, while anodal tDCS disrupted the adaptation. The effect of anodal and cathodal stimulation also seems to depend on the behavioral task that is considered. Bradnam et al. (2015) reported an overall positive effect of anodal cerebellar tDCS on handwriting with a reduced average pen pressure, but also a slower mean stroke frequency, which could indicate a worsening of the handwriting function. Interestingly, the same effects were found with cathodal tDCS for handwriting, but the improvements in cyclic drawing (decreased pen pressure, increased average speed) were only apparent for anodal tDCS.

These findings demonstrate that more information is required about the specific impacts of tDCS on cerebellar neurons to reliably predict the outcome of a given cerebellar stimulation.

### Current intensity, current density, and total charge

#### Current intensity

The study of Rampersad et al. (2014) showed that a cerebellar tDCS setup with the active electrode placed on the back of the head is accompanied by a large amount of shunting (see previous section). As a result, Rampersad et al. (2014) concluded that, in order to achieve comparable electric fields in the cerebellum, a larger input current has to be used (2 mA instead of 1 mA). Most of the studies with cerebellar tDCS have used an intensity of 2 mA, but several studies based on 1 or 1.5 mA also reported significant effects (Grimaldi and Manto, 2013; Shah et al., 2013; Dutta et al., 2014; Grimaldi et al., 2014b; Avila et al., 2015; Calzolari et al., 2015). Although a number of studies using cerebral tDCS found an effect of current intensity (Iyer et al., 2005; Boggio et al., 2006), Grimaldi and Manto (2013) failed to find a difference in stretch reflexes, upper limb coordination, or postural tests using two different current intensities for cerebellar tDCS (1 and 2 mA). Both intensities had a favorable effect on the amplitudes of the second stretch response (without changing the amplitude of the first stretch response) in cerebellar, but unfortunately no effect on upper limb coordination or posture (Grimaldi and Manto, 2013).

Experiments on cerebellar cells in animals have shown that an electric field between 1 and 20 V m^−1^ may interact with cerebellar neurons (Priori et al., 2014). Computational models of cerebellar tDCS using 2 mA predict an electric field in the cerebellum with a maximum ranging between 0.2 and 3.5 V m^−1^, which is consistent with the range of the predicted interaction with cerebellar neurons (Priori et al., 2014). The modeling study of Rampersad et al. (2014) using 1 mA, predicted a maximum electric field strength of 0.11 V m^−1^ of which 0.071 V m^−1^ was perpendicular to the gray matter surface, while Parazzini et al. (2014b) predicted an average value of ~1 V m^−1^. As a result, evidence from the modeling studies suggests that 2 mA might be needed for cerebellar stimulation to establish interaction with the cerebellar neurons. This is of course also dependent on the skin layer and the size of muscles of the neck. In animals, the skin and the muscles can be removed surgically to deliver directly the current over the skull or the dura, and therefore to obtain higher current densities (see also next section).

To deliver tDCS, it is important to use a device that can deliver a constant current (instead of a constant voltage) low resistance (Nitsche et al., 2003b). The use of conductive rubber saline-soaked sponges or a conductive gel is recommended (Nitsche et al., 2003b; Ferrucci et al., 2015). Brunoni et al. (2012) recommended solutions with relatively low NaCl concentration (between 15 and 140 mM) to reduce uncomfortable sensations during stimulation. These solutions require low voltage and allow for a good conduction of the current (Brunoni et al., 2012). Since tDCS devices are easy to construct with standard equipment numerous laboratories have designed their own device(s). A large variety of tDCS devices are thus used worldwide with several being commercially available. However, this lack of standardization of equipment represents a difficulty and even a major drawback to compare the results obtained in different studies (Brunoni et al., 2012).

#### Current density

Current density is determined by the intensity and by the surface area of the electrodes. For tDCS of the cerebellum, rectangular electrodes are typically used, measuring 5 × 5 cm (active electrode) or 5 × 7 cm (reference electrode; Ferrucci et al., 2015). To stimulate the cerebellum bilaterally a larger electrode (5 × 7 cm) is usually used. The larger the electrode, the larger the area stimulated and the smaller the current density. McCreery et al. (1990) showed that current densities below the limit of 25 mA/cm^2^ do not induce brain tissue damage. Since most studies with cerebellar tDCS report a current density of 0.08 mA/cm^2^, with a range from 0.057 to 1.3 mA/cm^2^ in the other studies, the current density remains well below the limit. Interestingly, Nitsche et al. (2003b) recommended to keep the current density below 0.02857 mA/cm^2^ (corresponding to 1 mA/35 cm^2^) since higher current densities might induce painful sensations. However, nearly all recent studies with tDCS report higher current densities and no painful sensations. Moreover, modeling studies indicate that within the cerebellar tissue a much lower current density is observed, even when the intensity of 2 mA is used (Parazzini et al., 2014b). Parazzini et al. (2014b) reported maximum current densities between 0.021 and 0.013 mA/cm^2^ in the cerebellum, depending on the model. The actual current density in the brain tissue depends largely on the resistance of the anatomical structures located above the target tissue. Resistance may vary from one person to another and should be taken into account when tDCS is used in subjects with skull defects or brain lesions, in subjects with neuropsychiatric disorders, or in subjects on pharmacotherapy (Brunoni et al., 2012). The rectangular shape of the sponges is somewhat arbitrary and have not been designed according to the anatomy of the skull.

#### Total charge

Total charge, which is determined by the duration of the tDCS sessions and the current intensity, is also an important factor in the procedure. Tissue damage has been observed for a total charge of 216 C/cm^2^ (Yuen et al., 1981). Few studies report the total charge, but since a stimulation of 15 min with a current of 2 mA intensity results in a total charge of 0.086 C/cm^2^ (Ferrucci et al., 2008), it is safe to assume that most studies remain well below the threshold, even when daily stimulation is applied over the course of several weeks.

### Sham procedure

To ensure that findings are really due to stimulation and not to a placebo effect or practice, the data are often compared with the measuring before and after a sham session. A sham session usually consists of ramping up the current to the same intensity as used in the active sessions, followed by immediately ramping down the current. In most studies this procedure results in an active stimulation of 30–60 s (Galea et al., 2009; Jayaram et al., 2012; Block and Celnik, 2013; Sadnicka et al., 2013; Chen et al., 2014; Dutta et al., 2014; Hardwick and Celnik, 2014; Herzfeld et al., 2014; Zuchowski et al., 2014; Benussi et al., 2015; Doeltgen et al., 2015; Martin et al., 2015; Panouillères et al., 2015a,b; Chothia et al., 2016; Van Wessel et al., 2016; Wessel et al., 2016). To effectively blind the subjects for sham or active stimulation, the electrodes remain on the head after ramping down the current to obtain the impression of comparable session lengths. Studies have shown that ramping down the current during cerebral stimulations does not elicit perceivable sensations, while sensations of turning on the current usually fade out in the first 30 s (Gandiga et al., 2006). As a result, sham sessions with at least 30 s of active stimulation with a ramping down of the current may effectively blind subjects (Russo et al., 2013). However, a study of Kessler et al. (2012) showed that the level of discomfort is higher during active sessions of tDCS than during sham sessions, making the implicit experience of the two conditions different. Kessler et al. (2012) speculated that the difference in discomfort is dependent on: (1) the duration of the ramp up and ramp down times, (2) the duration of the sham session, and (3) the intensity of the current. Adjusting these parameters might help to reduce the difference in (dis)comfort between sham and active stimulation. The following suggestions have been made:

1. Ramping up the current slowly instead of turning it on abruptly significantly reduces side-effects such as itching, shock sensation, or perception of a light flash (Nitsche et al., 2003a; Kessler et al., 2012). Since all studies included in the analysis of Kessler et al. (2012) used short ramp times of 10–15 s, the authors speculated that longer ramp times (~30 s) might result in similar sensations during active and sham tDCS. The tingling sensation at the beginning of the stimulation seems to be related to the increase of the current. Brunoni et al. (2012) demonstrated that a slow current increase of 0.1–0.2 mA/s does not generate any discomfort in most subjects.
2. The duration of the active stimulation during a sham session is important. Gandiga et al. (2006) reported comparable discomfort between sham and active sessions. This finding was probably due to the fact that the duration of the active stimulation during the sham session was increased to the mean duration of the sensations felt during active stimulation (Kessler et al., 2012). In addition, a minimum of 30 s of active stimulation in a sham session may cause skin redness beneath the electrodes. This reaction is typically induced by local vasodilatation after tDCS. Obtaining the same visual symptoms after sham sessions as after active stimulation (skin redness) is important to effectively mask sham from active stimulation for observers (Brunoni et al., 2012).
3. The total amount of charge delivered is dependent on the current intensity and the duration of the sessions and also influences the sensations felt during tDCS (Brunoni et al., 2012). Studies investigating the difference between cerebral sham and active tDCS have typically used current strengths of 1–1.5 mA (Gandiga et al., 2006; Poreisz et al., 2007; Kessler et al., 2012), but Kessler et al. (2012) predicted that using higher current intensities (such as 2 mA) could possibly make effective blinding difficult by exaggerating the difference in sensory side effects between sham and active stimulation. A study of O'Connell et al. (2012), in which a current strength was used of 2 mA during sessions of 20 min and sham stimulation of 30 s, found that participants were not adequately blinded with respect to the two conditions. However, O'Connell et al. (2012) used very short ramp up times of 5 s, resulting in a fast increase of current. This approach may have contributed to the poor blinding results. A study by Russo et al. (2013) also used current strengths of 2 mA and successfully achieved blinding results by using ramp up and ramp down times of 30 s and a longer stimulation of 90 s (30 s ramp up, 30 s stimulation at 2 mA, 30 s ramp down) during sham sessions, even though the total amount of charge delivered was even higher than in the study of O'Connell et al. (2012) (sessions of 30 min instead of 20 min).

Since current intensities of 2 mA are typically used in cerebellar tDCS, it seems important to use proper ramp times and maybe even a short period of active stimulation during sham sessions to ensure proper blinding of both the experimenter and the participant for a double-blind design. Practically, it may be recommended to use ramp times of a minimum of 30 s and to actively stimulate for at least 30 s during sham sessions, longer if the total amount of charge delivered is high.

### Sessions and duration

Multiple sessions are considered to have a cumulative effect and are needed to induce reliable and/or long-lasting after effects (Brunoni et al., 2012). The *repetition rate* seems to play a crucial role to induce cortical plasticity (Brunoni et al., 2012). In various cerebral stimulation trials, daily sessions have proved to be more effective than weekly sessions (Boggio et al., 2007) or sessions given every other day (Alonzo et al., 2012). In addition, Monte-Silva et al. (2010) showed that stimulating during ongoing after-effects of previous stimulation (during the MEP amplitude spike; 20 min break) resulted in prolonged and enhanced tDCS-induced effects. When the second stimulation was administered after remission of after-effects (normal MEP amplitude; 3 or 24 h break), the initial effects were first abolished or attenuated but then re-established after one (3 and 24 h break) to 2 h (24 h break only). Interestingly, no prolongation of the after-effects was observed if the stimulations were only 3 min apart. It has to be noted that this study only investigated cathodal tDCS in a healthy population. The optimal repetition rate and inter-stimulation interval has still to be determined for cortical tDCS (Brunoni et al., 2012).

Monte-Silva et al. (2010) also reported that *stimulation duration* (18 min instead of 9 min) has a beneficial impact on the duration of the after-effects. In comparison with a single session of 9 min, a single continuous stimulation of 18 min prolonged the after-effects from 60 to 90 min. However, this prolongation is less marked than the ones reported in a study of Nitsche et al. (2003c). These authors investigated the after effects of stimulation durations of 5, 7, and 9 min, indicating that there might be a ceiling effect of cathodal tDCS (Nitsche et al., 2003c).

The *number of repetitions* remains a matter of debate as well. Lindenberg et al. (2012) examined the effects of two 5-day intervention periods of bihemispheric cortical tDCS in a patient group. They showed that the second 5-day intervention also resulted in an increase of motor function, but a significantly lower one than after the first 5-day intervention. Therefore, repetitive sessions of tDCS do not necessarily induce a linearly cumulative result.

With regard to cerebellar tDCS, we lack systematic studies in which the effect of multiple sessions is studied or in which the effect of stimulation duration on cortical excitability is investigated. Most studies employing cerebellar tDCS have used a single session of 15–25 min of stimulation and reported variable outcomes (Ferrucci et al., 2015). The few studies based on multiple sessions are clinical studies. Most of these studies used multiple 5-day interventions (1–4 weeks) and administered one (Gironell et al., 2014; Ho et al., 2014; Minichino et al., 2015; Ferrucci et al., 2016) or two stimulation sessions a day (Bation et al., 2016). Bradnam et al. (2014), on the other hand, stimulated twice a week for a period of 12 weeks. The patients' conditions and outcome measures varied greatly across these studies.

Studies based on the same study populations to examine the effects of anodal, cathodal, and sham cerebellar tDCS usually separate the different stimulation conditions by at least 3 days (usually 1 week), with only two exceptions (1 day: Cantarero et al., 2015; 48 h: Foerster et al., 2013). Another way to avoid cross-over effects from previous stimulations is to start the experiment with the sham condition (Grimaldi et al., 2014b). Since long-lasting effects of tDCS-induced cerebellar excitability have not been investigated, it is recommended to adhere to long inter-session intervals (several days) or to use different groups of subjects/patients.

### Online vs. offline

tDCS can be administered in two different conditions: online or offline. If the effects of tDCS are measured during the application of tDCS, or if tDCS is administered simultaneously with another intervention (such as physical/cognitive therapy, or a training session), the study applies an online approach. An offline protocol uses tDCS in between two measurements without any practice and/or therapy during the stimulation (Brunoni et al., 2012). Because of an increased cortical excitability during stimulation, the online approach may be of utmost importance in rehabilitation settings of patients with neurological conditions (Priori et al., 2009). One of the major advantages of tDCS is that the mobility of the patient is unaffected during stimulation, making online application very interesting (Priori et al., 2009). However, the precise effect of online vs. offline stimulation has not yet been studied in detail (Monti et al., 2013) and is probably dependent on the intended outcome and the targeted area (Pirulli et al., 2013). Pirulli et al. (2013) tested the effect of timing of tDCS on the outcome in a visuo-perceptual learning experiment (stimulating the visual cortex). They found that, in contrast to motor learning (Nitsche et al., 2003d; Kuo, 2007; Stagg and Nitsche, 2011), offline administration of tDCS induces a greater effect than online stimulation, suggesting that the timing of tDCS has to be investigated for each area separately.

The literature is only scantly documented with regard to the difference between online and offline stimulation in cerebellar tDCS. One study used both online and offline cerebellar tDCS in two different experiments and showed that both applications may induce cortical excitability changes (Dutta et al., 2014). Cerebellar tDCS studies using an online protocol applied tDCS during a variety of tasks: in the adaptation phase of a motor learning protocol (Galea et al., 2009; Jayaram et al., 2012; Block and Celnik, 2013; Hardwick and Celnik, 2014; Herzfeld et al., 2014; Avila et al., 2015; Calzolari et al., 2015; Panouillères et al., 2015a,b; Yavari et al., 2016) during the learning/(mental) practicing of a task (Foerster et al., 2013; Shah et al., 2013; Dutta et al., 2014; Cantarero et al., 2015; Van Wessel et al., 2016; Wessel et al., 2016) or during the acquisition phase of a conditioned response (Zuchowski et al., 2014). Most studies, however, used an offline application of cerebellar tDCS, especially when clinical study populations were involved (Grimaldi and Manto, 2013; Bradnam et al., 2014; Gironell et al., 2014; Grimaldi et al., 2014b; Ho et al., 2014; Benussi et al., 2015; Bersani et al., 2015; Bradnam et al., 2015; Minichino et al., 2015; Bation et al., 2016; Ferrucci et al., 2016). In only one study, performed by Calzolari et al. (2015), online cerebellar stimulation was used in a single patient, applying cerebellar tDCS during a prism adaptation task. Martin et al. (2015), on the other hand, used online measurements to assess the effect of cathodal stimulation of the right cerebellum on working memory in patients with bipolar disorders.

Future studies are needed to define the effect of online and offline cerebellar tDCS. The efficiency of tDCS strongly depends on the timing of tDCS and may vary depending on the stimulated area (Pirulli et al., 2013). Therefore, the mechanisms by which tDCS affects cerebellar neurons, a critical step as already mentioned, have to be identified to address these issues properly.

### Effect of age and gender

Modeling studies based on models of different gender and age have demonstrated that current density distributions vary among individuals according to anatomy (Parazzini et al., 2014b). As clearly established in case of cerebral cortical tDCS, the subject's response to stimulation may also depend on age, gender, brain state, hormonal levels, and pre-existing regional excitability (Kuo, 2007; Krause and Cohen Kadosh, 2014). These factors should be taken into account when comparing different studies.

## Transcranial alternating current stimulation (tACS): a novel tool

Two studies using electrical stimulation of the cerebellum employed transcranial Alternating Current Stimulation (tACS) instead of tDCS (Mehta et al., 2014; Naro et al., 2016). tACS is an electrical stimulation technique that uses alternating currents in a given frequency range to stimulate the brain. Mehta et al. (2014) and Naro et al. (2016) have used the most frequently applied type of tACS consisting of a current intensity oscillating in a sinusoidal manner, going up and down in time to affect intrinsic cortical oscillations (Cohen Kadosh, 2013). Mehta et al. (2014) have investigated the effect of cerebellar tACS (2 mA, during tasks) on physiological tremor. The stimulating electrode was placed 3 cm right to the inion and the reference electrode on the contralateral shoulder. The frequency of the sinusoidal oscillating current was matched to each participant's task-dependent peak tremor frequency. Their study showed that cerebellar tACS increased entrainment of postural and kinetic tremor. Naro et al. (2016) attempted to modulate cerebellocerebral connectivity (fronto-parietal network) in patients with unresponsive wakefulness syndrome by means of cerebellar sinusoidally oscillating tACS (5 Hz, 2 mA, 10 min). The anode was placed over the medial cerebellum (half a centimeter below the inion) and the reference electrode over the left buccinator muscle. Sham sessions consisted of a 30 s active stimulation. Their study showed that cerebellar oscillatory tACS modifies functional connectivity within the fronto-parietal network, making tACS an interesting technique to study cerebellocerebral connections and interactions.

tACS can also consist of pulses of unidirectional current, rapidly increasing the current to the required intensity and dropping it back to zero after a short period of time several times in a row (Cohen Kadosh, 2013). The underlying effects of tACS are still unclear, but it is believed that it affects brain oscillatory activity. At low frequencies, the membrane potential changes in accordance with the current wave used in the stimulation (Deans et al., 2007). This leads to an alternating increase and decrease in neuronal excitability (Radman et al., 2007). However, due to the capacitive properties of the cellular membrane, this latter acts as a low-pass filter, which will tend to neutralize the effects of tACS at high frequencies. Bikson et al. (2004) reported that membrane polarization has a time constant over 10 ms (ranging from 14 to 70 ms). This means that neuron polarization by an electric field has a transient phase and is not immediate. Therefore, neurons will be less sensitive to fast alternating current (i.e., frequencies over 15 Hz; Bikson et al., 2004).

Several studies with cerebral tACS showed that the place of the reference electrode may influence the behavioral effect of the stimulation (e.g., Mehta et al., 2015). Mehta et al. (2015) tested four different montages with the stimulating electrode over the primary motor cortex, and the reference electrode varying between two cephalic (fronto-orbital and contralateral primary motor cortex) and two extra-cephalic (ipsilateral and contralateral shoulder) positions. They found that only the montage with the contralateral extracephalic electrode had a significant impact. This study suggests that the effects of tACS, as opposed to cerebellar tDCS (Grimaldi and Manto, 2013), critically depend on the electrode montage. On the other hand, the after effects of tACS seem to be induced by mechanisms similar to those seen in tDCS and might result from synaptic changes due to long-term potentiation and long-term depression of synaptic transmission (Nardone et al., 2015). However, more research is needed to clarify a number of unsolved issues related to tACS.

## General overview on cerebellar tDCS

tDCS is a promising electrical stimulation technique. By stimulating the cerebellum, modulation of cerebral cortical functions are achieved via the cerebellocerebral connections, using the cerebellum as a window to the whole brain as summarized by Priori (2014). However, little is known about the exact mechanisms by which tDCS modulates neuronal excitability of cerebellar modules. Due to its unique cytoarchitecture and the numerous motor and non-motor neurophysiological functions subserved by the cerebellum, these mechanisms might be difficult to establish (Schlerf et al., 2014). Studies systematically investigating the impact of tDCS on the various cerebellar neurons and functions are needed to define an efficient use of cerebellar tDCS in healthy and clinical populations.

Several studies have already specifically addressed the safety of applying currents of different densities and strengths to the brain. Based on a critical review of the literature, Nitsche et al. (2003b) have made recommendations, which are summarized in Table 2.

**Table 2:** **Recommendations regarding safety for application of tDCS in human (Nitsche et al., 2003b)**.

Use of non-metallic, conductive rubber electrodes covered completely by saline-soaked sponges
Maximum current density of 0.02857 mA/cm2
Maximum total charge of 0.022 C/cm2
Wedge-shaped on and off-current switch
Avoiding electrode montages that might cause brainstem or heart nerve stimulation
Stimulation device delivering a constant current density
Caution for stimulation above foramina (current can be focused)
Stimulation duration causing excitability changes >1 h should be applied cautiously in healthy subjects
Long-term excitability changes should not be induced more than once a week

Based on a critical survey of the available literature regarding cerebellar tDCS, some practical guidelines may be proposed. First, the *placement of the electrodes* used for the moment (rectangular sponges) does not seem to have a critical impact on the effects of cerebellar tDCS, provided the electrodes are positioned over the cerebellum. Therefore, it is not obligatory to use neuronavigation to determine the exact location of the active electrode (Parazzini et al., 2014b). There is no consensus for the location of the reference electrode (Grimaldi and Manto, 2013). In the majority of experimental set-ups, the reference electrode is positioned over the ipsilateral buccinator muscle. Both bilateral and unilateral setups have been studied. Second, in cerebral cortical stimulation settings, a consensus exists that *anodal* stimulation usually enhances cortical excitability, while *cathodal* stimulation inhibits excitability (Rahman et al., 2013). However, this is not a general rule for the cerebellum. Most authors consider that anodal tDCS of the cerebellum enhances the excitability of the cerebellar cortex whereas cathodal stimulation exerts opposite effects. Increased excitability of the cerebellar cortex will result in an increased inhibition of the cerebellar nuclei. Due to its complex folding and the specific arrangement of cerebellar micro-circuits, it remains hard to predict the effects of cerebellar tDCS on the various neuronal populations of the cerebellum and on each lobule (Woods et al., 2016). Most studies have investigated the effects of both anodal and cathodal tDCS in a great number of experimental protocols, and the outcomes of these studies have varied greatly. It is advised to use both *types* of stimulation if possible in cross-over designs or in different large groups of patients presenting the same disorder with a similar severity. Third, modeling studies showed that cerebellar tDCS causes a lot of shunting due to the placement of the electrode at the back of the head. It is therefore recommended to use a *current intensity* of at least 1.5 mA to evoke an interaction with the cerebellar neurons. Studies with smaller current intensities (1 mA) have reported effects of cerebellar tDCS, but most studies have used 2 mA. Fourth, in the majority of cases electrodes of 5 × 5 cm and/or 5 × 7 cm are used. These sizes ensure that the *current density* remains well below the recommended limit when a current intensity of 2 mA is used. Fifth, the *total charge* is dependent on the current intensity and on the duration of the stimulation. Most cerebellar tDCS studies used a single session of 15–25 min, remaining well below the recommended limit for total charge. As a result, it seems that multiple sessions of cerebellar tDCS can be applied safely. Sixth, it is recommended to use *sham* sessions and compare the results with the active stimulation sessions. Ramping up the current slowly instead of turning it on abruptly reduces the risk of (unpleasant) sensations (Nitsche et al., 2003a; Kessler et al., 2012). To ensure effective blinding ramp times of 30 s or longer are advised. Using at least 30 s of active stimulation during sham stimulation might therefore effectively blind the observer (Brunoni et al., 2012). Seventh, studies on *repetition rate, session duration*, and *number of sessions* have not been performed for cerebellar tDCS. Based on the findings of studies involving cerebral cortical tDCS, one or two 5-day interventions (Lindenberg et al., 2012), with daily stimulation (Brunoni et al., 2012) of ~20 min (Monte-Silva et al., 2010) might be suggested. Whether this protocol is also relevant for cerebellar neurostimulation still remains a matter of debate. A similar observation holds for *online or offline* application of cerebellar tDCS. Cerebral cortical tDCS studies have demonstrated that the effect of online or offline application is not straightforward and depends on the stimulated area (Pirulli et al., 2013). What the effect of both types of applications are on the cerebellum remains to be elucidated. Eighth, *tACS* is a novel technique that is believed to impact cerebral cortical oscillations. Naro et al. (2016) showed that this type of noninvasive electrical stimulation may be very promising to study cerebellocerebral connections. tACS seems more sensitive to electrode placement than tDCS and even less is known about the effects of tACS on cerebral and cerebellar neurons and the mechanisms by which it modulates neuronal excitability.

## Conclusion

Both tDCS and tACS are promising novel non-invasive electrical stimulation techniques to modulate cerebellar function. Since the cerebellum is considered as a window to modulate the function of distant cortical regions via reciprocal cerebellocerebral loops, tDCS and tACS have a strong potential that should be explored in detail in future research. Systematic studies investigating the impact of different setups and protocols are needed to elucidate the exact mechanisms by which these types of electrical stimulation influence cerebellar excitability, both at the level of the cerebellar cortex and cerebellar nuclei. The short-term and long-term effects on the olivo-cerebellar pathways and the mossy fiber pathways are currently unknown. It is also unclear how tDCS and tACS modulate the activity of the nucleo-olivary tracts acting upon the inferior olivary complex, the discharges of the parallel fibers, the activity of the nucleo-cortical loops or the activity of the spinal cord circuits.

## Author contributions

KVD, FB, PM, MM reviewed the literature under the coordination of KVD who selected the articles. KVD and FB drafted the first version of the manuscript. PM and MM corrected the draft. KVD, FB, PM, MM have read and approved the final version.

### Conflict of interest statement

The authors declare that the research was conducted in the absence of any commercial or financial relationships that could be construed as a potential conflict of interest.
